# Temperature Down‐Shift Modifies Expression of UPR‐/ERAD‐Related Genes and Enhances Production of a Chimeric Fusion Protein in CHO Cells

**DOI:** 10.1002/biot.202000081

**Published:** 2020-04-27

**Authors:** Mauro Torres, Samia Akhtar, Edward A. McKenzie, Alan J. Dickson

**Affiliations:** ^1^ Manchester Institute of Biotechnology Faculty of Science and Engineering University of Manchester Manchester M1 7DN UK; ^2^ Protein Expression Facility Manchester Institute of Biotechnology Faculty of Life Sciences University of Manchester Manchester M1 7DN UK

**Keywords:** biopharmaceuticals, Chinese hamster ovary cells, low temperature stress, transcriptome, unfolded protein response/endoplasmic reticulum associated degradation

## Abstract

Low culture temperature enhances the cell‐specific productivity of Chinese hamster ovary (CHO) cells expressing varied recombinant (r‐) proteins, but the mechanisms remain unclear. Regulation of unfolded protein response (UPR) pathway genes, such as transcriptional regulatory factors and endoplasmic reticulum (ER)‐resident proteins, appear to be involved in the improvements of r‐protein production under low temperature conditions. The transcriptional regulation of UPR‐specific targets is studied in response to decreased culture temperature in relation to production of a difficult‐to‐express protein. A clonally‐derived CHO cell line expressing a chimeric fusion protein (human erythropoietin [hEPO] linked to a murine Fc region, hEPO‐Fc) is evaluated in terms of growth, metabolism, r‐protein production and UPR‐/ER associated degradation (ERAD)‐specific gene expression at standard (37 °C) and low (32 °C) temperature in batch and fed‐batch systems. Low temperature decreased peak cell density, improved viability, generated cell cycle arrest in the G1 phase and enhanced hEPO‐Fc expression in both batch and fed‐batch cultures. A low culture temperature significantly upregulated genes encoding UPR‐specific transcriptional activators (*xbp1s*, *ddit3*, and *atf5*) and ER‐resident proteins (*grp78*, *grp94*, *trib3*, and *ero1α*), that are associated with folding and processing of proteins within the ER. Further, low culture temperature decreased expression of genes involved in ERAD (*edem3*, *sels*, *herpud1*, and *syvn1*) indicating a decreased potential for protein degradation.

## Graphical abstract

Decreased growth rate and increased specific productivity of a recombinant Chinese Hamster Ovary cell line (secreting a model Fc‐fusion protein) due to lowered culture temperature (from 37 °C to 32 °C) is associated with selective changes to mRNA species encoding key proteins in the unfolded protein response (upregulation) and endoplasmic reticulum‐associated degradation pathways, providing an integrated control model for enhanced specific productivity.

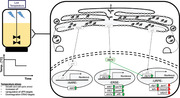

## 1 Introduction

Chinese hamster ovary (CHO) cell lines are the most used expression platform for the manufacture of commercial therapeutic recombinant (r‐) proteins at an industrial scale (84% of the mAbs approved from 2015 to 2018).^[^ ^1^ ^]^ Although improvements both in CHO cell line development and media over recent years have led to highly productive bioprocesses,^[^ ^2^ ^]^ the expression for some r‐proteins, particularly those with more complex biological formats (e.g., multi‐specific antibodies or fusion proteins), remains challenging. Limitations in the secretory pathway, particularly during the protein processing in endoplasmic reticulum (ER), have been proposed to be the main bottleneck for the production of secreted r‐proteins in CHO cells.^[^ ^3^ ^]^ These cell lines present an increased ER protein folding demand that often exceeds their protein folding capacity, which results in the accumulation of unfolded/misfolded proteins and ER stress.^[^ ^4^ ^]^ Such a scenario has been observed in many stable CHO cell lines expressing different r‐proteins (e.g., monoclonal antibodies and Fc fusion proteins) and is particularly exacerbated in those cell lines expressing “difficult‐to‐express” (DTE) protein formats.^[^ ^3^, ^5^, ^6^, ^7^, ^8^, ^9^, ^10^, ^11^, ^12^ ^]^ In this context, culture environment has a significant impact on production of secreted r‐proteins in CHO cell systems, and physiological changes that arise from optimizing culture conditions may increase our understanding of the component interplay in the secretory pathways. Culture temperature, particularly, has been largely studied and applied in the context of CHO cell‐based r‐protein production since a decrease in a few degrees (from the standard 37 °C to 35–30 °C) has enhanced cell‐specific productivity in several cell lines with varied r‐protein models.^[^ ^13^, ^14^, ^15^, ^16^ ^]^ Although the reasons behind this enhanced r‐protein production are still unknown, researchers/studies have suggested a long list of possible explanations, such as extended viability (and culture lifespan), decreased apoptosis,^[^ ^17^ ^]^ better metabolic efficiency,^[^ ^16^ ^]^ arrested cell cycle in G1/G0 (so‐called “productive phase”),^[^ ^18^ ^]^ improved transcription and stability of the r‐gene^[^ ^19^ ^]^ and enhanced secretory capacity.^[^ ^20^ ^]^

Recent transcriptomic and proteomic analyses have unveiled physiological insights related to CHO cell performance under moderate hypothermic conditions. An RNA‐seq analysis of a r‐CHO cell line expressing ht‐PA that underwent a temperature down‐shift (TDS) showed a significant upregulation of several genes coding for ER chaperones, transmembrane ER channels and other folding and trafficking‐associated proteins, whilst a downregulation was observed for genes involved in N‐glycosylation and folding sensor ER quality control function.^[^ ^21^ ^]^ A similar increased gene expression of protein‐folding chaperones and an ER resident transmembrane sensor (PERK) was also observed in two different CHO cell lines expressing mAbs after TDS using DNA microarrays.^[^ ^22^, ^23^ ^]^ mRNA expression of the spliced form of X‐box binding protein 1 (XBP1s) was evaluated in two CHO cell lines with different r‐protein productivities (i.e., low and high producers) using quantitative PCR (qPCR). Greater XBP1s mRNA abundance was found in high producing clones compared to the low producers at low temperature and this was correlated to specific r‐protein productivity.^[^ ^16^ ^]^ Decreased culture temperature also improved folding and secretion of a human tumor necrosis factor receptor II fusion protein (TNFR‐Fc) in CHO cells, partly by decreasing aggregation in a PERK‐dependent mechanism.^[^ ^24^ ^]^ In this context, we hypothesize that enhanced cell‐specific producitivities of CHO cells (at low temperature) are associated with upregulation of ER‐resident proteins and unfolded protein response (UPR)‐specific transcriptional factors that improve the protein folding capacity of cells and lead to increased product titers.

In this study, we investigated the effect of low temperature on the expression of genes involved in the secretory and ER‐associated protein degradation (ERAD) pathways in a r‐CHO cell line expressing a DTE protein model. To address this objective, we generated a CHO cell line expressing a chimeric fusion protein consisting of human EPO linked to a murine Fc region (hEPO‐Fc), isolated a clone based on productivity and evaluated its culture performance (proliferation and r‐protein expression) at low (32 °C) and standard (37 °C) temperatures in both batch and fed‐batch systems. The gene expression profile of a set of 15 UPR/ERAD‐related genes (involved in protein sensing, processing, folding and degradation) to low temperature was evaluated throughout culture, with a focus on early, mid, and late exponential phases. Regulation of the secretory pathways in CHO cell systems has been previously investigated using chemical additives, such as tunicamycin (TM, an N‐glycosylation inhibitor), that induce ER stress and alter the gene expression of UPR‐specific targets.^[^ ^25^, ^26^ ^]^ In the present study, our CHO cells were treated with TM as an ER stress control and the gene expression pattern was compared between chemically‐induced and temperature‐induced stress. Our results indicated that low temperature enhanced protein processing and folding capacity of CHO cells by upregulation of genes involved in the IRE1/XBP1 branch of the UPR, and parallel downregulation of ERAD machinery suggesting that manipulation of these pathways presents potential targets for cell engineering to enhance protein secretion of DTE proteins.

## 2 Experimental Section

### 2.1 Plasmid Design and Cell Line Generation

The plasmid expressing a chimeric recombinant fusion protein was constructed by sub‐cloning human erythropoietin (hEPO) and a murine immunoglobulin gamma 2 Fc region (mIgG2a Fc) sequences into the *HindIII* and *XhoI* sites of pcDNA3.1 vector (Invitrogen), under the control of a cytomegalovirus (CMV) promoter, with neomycin as the selection marker under the control of a SV40 promoter. Both the hEPO and the mIgG2a Fc domains were codon‐optimized in silico for CHO cells using SnapGene software (version 4.1.7., GSL Biotech LLC). The hEPO sequence was modified by removing the C‐terminal arginine to avoid the loss of the Fc domain during the maturation process of the r‐protein.^[^ ^27^ ^]^ A C‐terminal 6×His tag was added to the murine mIgG2a domains to facilitate affinity purification. The plasmid was purified using a Qiagen Plasmid Maxi Kit (QIAGEN) and linearized using PvuI‐HF restriction enzyme (NEB) prior to transfection. A suspension‐adapted CHO‐K1 cell line was used as a host for expressing the hEPO‐Fc fusion protein. The expression plasmid was introduced into the host cell line using a standard electroporation method. Prior to electroporation, 1.2 × 10^7^ cells in exponential growth with >95% viability were washed in cold 1×PBS and mixed with 30 µg of the linearized DNA plasmid in a total of 1 mL of sterile 1×PBS. Two consecutive pulses (300 volts, 950 µF, and 20 ms) were delivered to the mixture in an electroporation cuvette (Bio‐Rad) using a Bio‐Rad Gene Pulser XCell system (Bio‐Rad). Cells were then placed on ice for 5 min and resuspended in CD FortiCHO medium (Gibco) supplemented with 4 mm L‐glutamine (Life Technologies) and 1% (v/v) Geneticin (G418, Gibco) as selection medium. Cells were subjected to limiting dilution cloning according to Barnes et al.^[^ ^28^ ^]^. Wells containing single colonies were expanded from 96 to 24 well plates, then on to T‐25 and T‐75 static flasks. The top hEPO‐Fc producer (CHO‐hEPO‐Fc) determined by enzyme‐linked immunosorbent assay (ELISA) and western blot was scaled‐up to 125 mL shake flask culture.

### 2.2 Cell Culture and Media

The CHO‐hEPO‐Fc cell line was cultured in CD FortiCHO medium supplemented with 4 mm L‐glutamine and 1% (v/v) G418. Maintenance cultures were seeded at 2 × 10^5^ cells mL^−1^ and were sub‐cultured every 3 days with fresh medium in 50 mL mini‐bioreactors (Corning) at a working volume of 10 mL. All cultures were maintained at 37 °C with shaking at 230 rpm, 5% CO_2_‐enriched atmosphere and 90% relative humidity in a ThermoForma Reach‐In CO2 incubator (Thermo Fisher). Batch (BC) and fed‐batch (FB) cultures were performed in 250 mL vented‐cap Erlenmeyer shake flasks (Corning) with a working volume of 50 mL and at a starting cell density of 2 × 10^5^ cells mL^−1^. Culture medium for BC and FB cultures was the same as that used for maintenance cultures. For FB cultures, the feeding regime consisted of 4 additions (2.5 mL) of feed medium added at days 2, 4, 6, and 8 of culture (the last feed was only added for 32 °C cultures). The customized feed consisted of glucose (50 mm), glutamine (5 mm), asparagine (2.5 mm), serine (2.5 mm), and pyruvate (5 mm). The feed was prepared by dissolving the components in milliQ water and by sterilization through 0.22 µm filters. For low temperature cultures, BC and FB cultures were switched to 32 °C (bi‐phasic cultures) after the initial 48 h with control cultures maintained at 37 °C. Cell cultures were performed in triplicate (biological replicates) for all conditions. Sampling for cell counting, metabolite analysis, cell cycle analysis, and mRNA expression analysis was carried out for all cultures every 24 h until viability decreased to 70%. For tunicamycin‐treated cultures, the CHO‐hEPO‐Fc cell line was cultured under batch conditions (as described earlier), and after 3 days (≈2 × 10^6^ cells mL^−1^) supplemented with TM (6 µg mL^−1^). Control cultures were maintained without TM in parallel. Sampling for RNA was performed at 6 h after TM treatment. Cell concentration and viability were determined by haemocytometer analysis (Neubauer, Germany) using the trypan blue exclusion method. Specific growth rate (µ) was determined as previously described in Torres et al.^[^ ^16^ ^]^

### 2.3 Cell Cycle Analysis

For cell cycle analysis, 1.5 × 10^6^ cells in 200 µL PBS were fixed with 1.8 mL cold 70% (v/v) ethanol and samples were stored at −20 °C until measurement. Fixed cells were mixed with 1 mL propidium iodide solution (40 µg mL^−1^ final concentration, Invitrogen, USA) in 3.8 mm sodium citrate (Sigma, USA). After an overnight incubation at 4 °C with 50 µL stock RNase A solution in the dark, cell cycle status was determined using a BD FACSCanto‐II flow cytometer (BD Biosciences, USA) and data acquisition was performed using BD FACSDiva software (BD Biosciences, USA). Flow cytometry data was analyzed using FlowJo software (FlowJo, LLC, USA). Results were presented as the percentage of cells in G1/G0, S, and M phases of the cell cycle.

### 2.4 Analysis of Recombinant hEPO‐Fc Expression

#### 2.4.1 ELISA of Secreted hEPO‐Fc

Secreted hEPO‐Fc concentration was quantified using an in‐house ELISA method. Briefly, ELISA plates (# 439454, Thermo Fisher Scientific, USA) were coated with 0.5 µg mL^−1^ of AffiniPure Goat Anti‐Mouse IgG‐Fc antibody (#115‐005‐008, Jackson ImmunoResearch) in phosphate‐buffered saline (PBS, 137 mm NaCl, 2.7 mm KCl, 10 mm Na_2_HPO_4_, 2 mm KH_2_PO_4_, pH 7.4) at 4 °C for 16 h. The plates were incubated in blocking buffer (PBS with 0.1% [v/v] Tween‐20 [PBS‐T] and 1% [w/v] BSA) at RT for 1 h. After each step, plate wells were washed extensively with PBS‐T. Culture medium and hEPO‐Fc standards (diluted with PBS‐T/BSA) were added to the wells and the plates were incubated at RT for 1 h. Bound hEPO‐Fc was detected using a rabbit antibody against human‐EPO (mAb286, R&D Systems, USA) at 0.4 µg mL^−1^ followed by a goat anti‐Rabit IgG H+L (HRP) secondary antibody (#31466, Invitrogen, USA) at 0.25 µg mL^−1^. Reactions were visualized using TMB substrate solution. 2 m H_2_SO_4_ solution was added to stop the reaction and the absorbance for each well was measured at 450 nm using a Varioskan LUX microplate reader (Thermo Scientific). Data acquisition was performed with SkanIt software (Thermo Scientific). Sample concentrations were determined using standard curves generated by linear regression. hEPO‐Fc was purified from culture supernatant using protein A agarose beads (Roche) and used as a standard. Specific hEPO‐Fc productivity (*q*_hEPO‐Fc_) and overall product yield (*Y*_hEPO‐Fc_) were determined as previously described in Torres et al.^[^ ^16^ ^]^

#### 2.4.2 Western Blot Analysis of Intracellular and Secreted hEPO‐Fc

For western blot analysis, 1 × 10^7^ viable cells were harvested by centrifugation (500 g, 10 min) and culture supernatant were collected and resolved by SDS/PAGE as described previously.^[^ ^7^ ^]^ Proteins separated by SDS–PAGE were transferred onto a nitrocellulose membrane using the Bio‐Rad Semi‐Dry transfer system according to the manufacturer's instructions. Membranes were blocked in 5% (w/v) milk in PBS‐T for 1 h at RT. Membranes were incubated for 1 h with a IRDye 800CW Goat anti‐Mouse (926‐32210, LI‐COR Biosciences) as primary antibody. Proteins were detected using a ChemiDoc MP Imaging System (Bio‐Rad) according to the manufacturer's instructions and data acquisition was performed with Image Lab software (version 6.0.1., Bio‐Rad).

### 2.5 Gene Expression Analysis by PCR and q‐RTPCR

For mRNA expression analysis, 1 × 10^7^ viable cells were harvested by centrifugation (500 g, 10 min) at day 3 and 6 in BC cultures and at day 3, 6, and 9 in FB cultures, both at 37 °C and 32 °C. After centrifugation, cell pellets were lysed in 1 mL Trizol Reagent (Invitrogen) and processed according to the manufacturer's instructions. The nucleic acid concentration and purity were determined using a Nanodrop ND‐1000 UV–vis spectrophotometer (Thermo Fisher Scientific). RNA sample quality was assessed and an A260/A280 ratio between 1.8 and 2.0 and an A260/A230 ratio >2.0 were regarded as appropriate for analysis. Genomic DNA was removed through treatment with DNase 1 (NEB) and the resulting RNA was converted to complementary DNA (cDNA) in a total reaction volume of 100 µL using Tetro cDNA synthesis kit (Bioline) as per the manufacturer's instructions using a TC‐3000 Thermal Cycler (Techne). cDNA samples and negative control samples (samples which had not been treated with reverse transcriptase) were prepared for RT‐PCR and RT‐qPCR by diluting in nucleic free water. PCR reactions were carried out in a total volume of 50 uL using a BIOTAQ DNA polymerase kit (Bioline) as per the manufacturer's instructions with primer (10 µm) and 1 µL cDNA. PCR was performed with the following TC 3000 X thermal cycler programme: 94 °C 5 min, then 27 cycles of 94 °C 30 s, annealing temperature at 57 °C for 30 s, 72 °C 30 s then 72 °C for 10 min. PCR primers are shown in Table S1, Supporting Information. Glyceraldehyde 3‐phosphate dehydrogenase (*gapdh*) and beta‐actin (*β‐actin*) housekeeping genes were used as positive controls. PCR products were resolved on 2% (w/v) agarose gels. RT‐qPCR reactions were performed by mixing 2 µL primers (10 µm) with 2.5 µL cDNA (1:2 in nuclease free water) sample and 5 µL SYBR Select Master Mix (Applied Biosystems, USA) in 96‐well plates. All samples were analyzed in triplicate. Additionally, standard curves based on a serial dilution from 1:1 to 1:32 of cDNA sample were performed to test the amplification efficiency of each primer. The qPCR reaction was performed using a QuantStudio 5 Real‐Time PCR System (Thermo Fisher Scientific, USA) and data acquisition was performed using QuantStudio Design & Analysis Software v1.4.2. (Thermo Fisher Scientific, USA). The thermal cycling parameters used consisted of an initial denaturation step of 2 min at 95 °C, followed by 40 cycles of 15 s at 95 °C and 15 s at 57 °C. A final extension of 60 s at 72 °C was performed, followed by a melting curve to confirm primer specificity. Data were analyzed using the 2‐ΔΔCt method and normalized using *gapdh* as standards.^[^ ^29^ ^]^

### 2.6 Statistical Analysis

Kinetic parameters were calculated from three biological replicates and were expressed as mean ± standard error (SEM). All statistical analyses were performed with R software (version 3.1.^[^ ^30^ ^]^). Significant differences in culture parameters and mRNA expression levels were analyzed by a Two‐way ANOVA and *t*‐test, as previously described.^[^ ^16^ ^]^ The threshold for statistical significance was *p* < 0.05.

## 3 Results

### 3.1 Analysis of Growth and Cell Cycle Analysis

**Figure 1:**
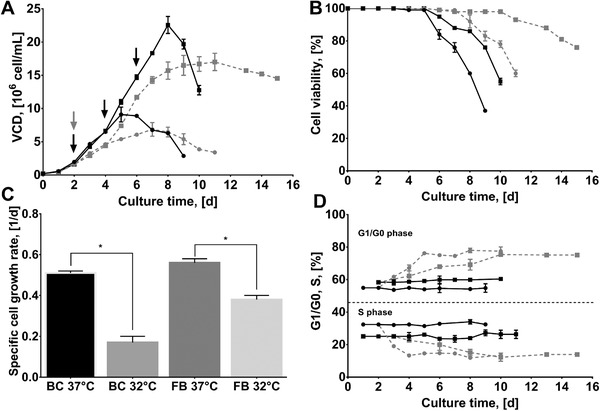
Growth, viability and cell cycle distribution profiles of CHO‐hEPO‐Fc cell line during batch (●) and fed‐batch (■) cultures at 37 °C (black) and 32 °C (grey). A) Viable cell densities (VCDs) throughout cultures. Black arrows indicates feeding time points for FB both at 37 °C and 32 °C, while a grey arrow corresponds to the temperature down‐shift point both for BC and FB. B) The percentage of viable cells throughout cultures. C) The specific cell growth rates (µs) of the CHO‐hEPO‐Fc cell line in each culture condition. The µs were calculated from day 2 to the day when cells reached VCDmax and “*” indicated *p* < 0.05 (*t*‐test). D) The percentage of cells in G1/G0 and S phases of the cell cycle. No significant variations in G2/M phase was observed. Experimental values represent the mean of three biological replicates and error bars indicate ± SEM.

The impact of culture temperature on cell growth and cell cycle distribution of the CHO‐hEPO‐Fc cell line was assessed in both BC and FB systems by shifting from 37 °C to low temperature (32 °C) after 48 h. Cultures were performed in triplicate and analyzed until cell viability decreased below 70%, a time when glucose in medium was completely depleted. As expected, culture temperature had a significant impact on cell growth and viability of the CHO‐hEPO‐Fc cell line in both BC and FB (**Figure** **1**) (Two‐way ANOVA, *p* < 0.05). Maximal viable cell densities (VCD_max_) and specific cell growth rates (µs) were reached at 37 °C, reaching 9.1 × 10^6^ cells mL^−1^ and 0.51 1per day in BCs and 22.6 × 10^6^ cells mL^−1^ and 0.56 per day in FCs. Low culture temperature decreased the VCD_max_ by 25% in BC (Tukey's post hoc test, *p* < 0.05) and by 18% in FB (*p* < 0.05) compared to the control cultures, while the specific growth rate was decreased by 67% in BC (*p* < 0.05) and by 32% in FB (*p* < 0.05) (Figure 1A,C). Cell viability declined from day 6 in both BC and FB at 37 °C, while at 32 °C the start of the decline phase was extended by 2 days in BC and by 5 days in FB (Figure 1B). These changes observed in cell growth and viability were in agreement with previous published studies in low temperature CHO cell cultures, where an overall decrease in peak of viable cell density and specific growth rate and improved cell viability and culture lifespan have been observed in different recombinant cell lines expressing varied model r‐proteins.^[^ ^16^, ^31^, ^32^, ^33^ ^]^

Cell cycle distribution was analyzed by calculating the percentage of cells in G1/G0, S, or M phases throughout the culture time for all conditions. The temperature downshift greatly affected cell cycle distribution, particularly G1/G0 and S phases (significant differences observed from day 6 onward, Two‐way ANOVA, *p* < 0.05) (Figure 1D). For cultures at 37 °C, the percentage of cells in G1/G0, S, and G2/M phases remained constant throughout the culture period, with a slight increase of the number of cells in G1/G0 at the late stage of cultures. Additionally, FB showed a greater number of cells in G1/G0 phase compared to BC and this increase was reflected in a decrease in the number of cells in S phase. For all cultures at 32 °C, the percentage of cells in G1/G0 phase progressively increased up to 75% (Figure 1D). In parallel, the percentage of cells in S phase decreased to 13% after the TDS. No significant variations were observed in the number of cells in G2/M phases during the culture time. Results regarding the cell cycle distribution of the CHO‐hEPO‐Fc cell line were consistent with previously reported data in CHO cell cultures, showing a cell cycle arrest in G1/G0 phase at low temperature (either by a rapid down‐shift or by acclimatization).^[^ ^32^, ^34^ ^]^ However, an increasing number of cells in G1/G0 phase corresponded to decreasing the number of cells in S phase and without affecting the G2/M phase distribution, a finding which has not been previously reported for CHO cell cultures. Additionally, we observed that the feeding regime did not impact the percentage of cells located in the G1/G0 phase at the end of the growth phase, although it increased the transition time by 2 days compared to BC at 32 °C (Figure 1D).

### 3.2 Expression of Recombinant hEPO‐Fc

**Figure 2:**
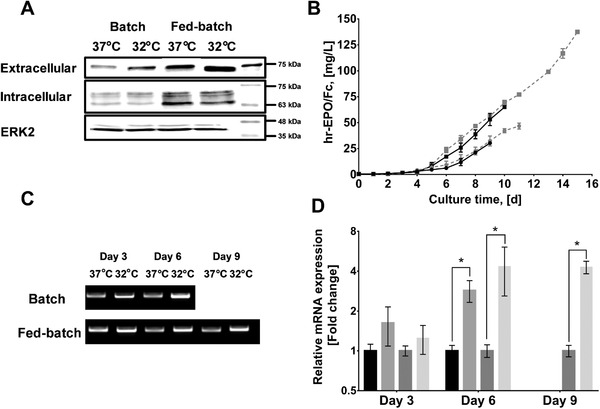
Expression of the r‐protein in the CHO‐hEPO‐Fc cell line during batch and fed‐batch cultures at 37 °C and 32 °C. A) Extra‐ and intracellular protein analysis using western blot. Samples for secreted protein correspond to day 8 (37 °C) and day 10 (32 °C) for BC, and to day 10 (37 °C) and day 14 (32 °C) for FB. ERK2 was used as internal standard for intracellular protein analysis to confirm that the amount of protein loaded in each lane was comparable among conditions. B) The secreted hEPO‐Fc profile during batch (●) and fed‐batch (■) cultures at 37 °C (*black*) and 32 °C (*grey*). C) The band intensity of PCR analysis of the r‐hEPO‐Fc. D) The relative mRNA expression analysis of r‐hEPO‐Fc, calculated using 2‐ΔΔCt method (with 32 °C and 37 °C as treatment and control conditions) and normalized to *gapdh*. The data represents BC at 37 °C (■), BC at 32 °C (■), FB at 37 °C (■), and FB at 32 °C (■) for day 3, 6, and 9 (only for FB culture). “*” indicated *p* < 0.05 (*t*‐test). Experimental values represent the mean of three biological replicates and error bars indicate ± SEM.

The expression of the recombinant hEPO‐Fc was monitored at both mRNA and protein level throughout the cultivation of the CHO‐hEPO‐Fc cell line (**Figure** **2**). The presence of secreted (extracellular) and intracellular hEPO‐Fc was determined by western blot analysis using an Fc‐domain detection antibody (Figure 2A). For secreted hEPO‐Fc, a single band of ≈75 kDa (with a slightly lower band size at 32 °C) was identified in all conditions and higher abundance of protein was found in cultures at 32 °C both in BC and FB systems. Identification of intracellular hEPO‐Fc showed three bands with different molecular weights, an upper (≈70 kDa), a mid (≈68 kDa), and a lower (≈65 kDa) bands, that represented partly‐glycosylated forms. While BC did not present variations in the intracellular levels of hEPO‐Fc, FB at 37 °C showed a higher amount of the lowest molecular weight form compared to 32 °C. An internal protein standard (ERK2) was also analyzed in western blot analysis to ensure that cells are producing similar amount of proteins at intracellular level. As expected, no detectable differences were observed in the ERK2 bands among the culture conditions were observed (Figure 2A). Therefore, no significant changes in the intracellular amounts of hEPO‐Fc was observed as an explanation to underpin the enhanced secreted protein at decreased culture temperature. Secreted hEPO‐Fc concentrations were measured in BC and FB cultures at both 37 °C and 32 °C (Figure 2B) and from these results, the specific production rates (*q*_hEPO‐Fc_) and overall production yields (*Y*_hEPO‐Fc_) were calculated at all conditions (**Table** **1**). The product titers were comparable among all cultures before and after the TDS up to day 5 and increased exponentially after day 5 to the end of culture. Low culture temperature significantly affected the peak of hEPO‐Fc concentration (Titer_max_), the *q*_hEPO‐Fc_ and the *Y*_hEPO‐Fc_ compared to the controls in both BC and FB cultures (Table 1) (two‐way ANOVA, *p* < 0.05). The TDS increased the Titer_max_ by 54% in BCs (Tukey's post hoc test, *p* < 0.05), by 111% in FB conditions (*p* < 0.05), while the *q*_hEPO‐Fc_ and the *Y*_hEPO‐Fc_ were increased by 49% (*p* < 0.05) and 27% (*p* < 0.05) in BC conditions, and by 58% (*p* < 0.05) and 42% (*p* < 0.05) in FB conditions, respectively. Increased *q*_hEPO‐Fc_s in low temperature cultures also coincided with a higher amount of the r‐gene (Figure 2C,D) and arrested cell cycle in G1/G0 phase (Figure 1D), while improved cell viability did not contribute to better productivities as the *q*_hEPO‐Fc_s were higher at 32 °C that 37 °C in both batch and fed‐batch at timepoints when the viability was 100% in every condition. The amount of hEPO‐Fc reported here was in the range of previous studies (from 0.02 mg L^−1[^ ^36^ ^]^ to 200 mg L^−1[^ ^37^ ^]^ under different culture conditions). In addition, the higher *q*_hEPO‐Fc_ and *Y*_hEPO‐Fc_ in low temperature cultures were consistent with previous reported data, indicating an increased r‐protein production and cell‐specific productivity due to a decrease in culture temperature.^[^ ^16^, ^31^, ^35^, ^36^ ^]^

**Table 1:** Culture parameters for hEPO‐Fc production in CHO cells during batch and fed‐batch cultures at 37 °C and 32 °C

Parameters	Unit	Batch			Fed‐batch		
		37 °C	32 °C	FC	37 °C	32 °C	FC
Titer max	mg L^−1^	30.4 ± 1.3	46.8 ± 2.8	1.5^*^	64.9 ± 1.6	137.5 ± 1.1	2.1^*^
*q*_hEPO‐Fc_	pg per cell day^−1^	0.70 ± 0.21	1.14 ± 0.29	1.6^*^	0.65 ± 0.11	1.13 ± 0.23	1.7^*^
*Y*_hEPO‐Fc_	mg L^−1^ day^−1^	3.4 ± 0.2	4.3 ± 0.3	1.3^*^	6.5 ± 0.2	9.2 ± 0.1	1.4^*^

* Indicates statistical significance (*p* < 0.05) using *t*‐test. Error bars represent ± SEM.

FC indicates the fold change value between 32 °C and 37 °C

Expression of recombinant hEPO‐Fc was also analyzed at transcriptional level using PCR and qPCR throughout cultures (Figure 2C,D). An increased expression of recombinant *hEPO‐Fc* mRNA was observed after TDS in BC and FB (Figure 2C). This observation was confirmed using qPCR analysis, calculating the ratio of the relative mRNA content of each gene with respect to *gapdh* (Figure 2D). TDS increased *hEPO‐Fc* mRNA expression up to fourfold by the end of the exponential phase (day 6 for BC and day 9 for FB), whereas no significant variations were observed in the relative mRNA expression in terms of culture mode (at 37 °C and 32 °C) (data not shown). Our results showed a time‐dependent response of r‐gene expression to decreased temperature, with fully impact observed after 3 days. In previous publications, a temperature decrease in CHO cells increased the amounts of r‐gene mRNA by a mechanism associated with enhanced mRNA stability^[^ ^20^ ^]^ as well via increased transcriptional activity.^[^ ^37^ ^]^ In our studies we have not distinguished if changes to transcript amounts (r‐gene RNA or other targets) arises as a result of changes to mRNA production or stability in response to low temperature culture. This caveat applies to the mechanisms responsible for changes to all transcripts measured in this study. This response is also relevant for low temperature‐focused transcriptomic studies, which often are analyzed 24 or 48 h after the temperature shift. We analyzed the relationship between *hEPO‐*Fc mRNA expression and *q*_hEPO‐Fc_ in order to determine whether secreted r‐protein rates were determined by the amount of transcript. However, there was no strong correlation between r‐hEPO‐Fc mRNA and *q*_hEPO‐Fc_ (Pearson's correlation = 0.52, *p* = 0.078) (Figure S1, Supporting Information), which would suggest that post‐transcriptional events dominate the extent of production and secretion of hEPO‐Fc in response to TDS.^[^ ^3^ ^]^

### 3.3 Expression Profile of Genes Involved in the UPR and ERAD Pathways

**Figure 3:**
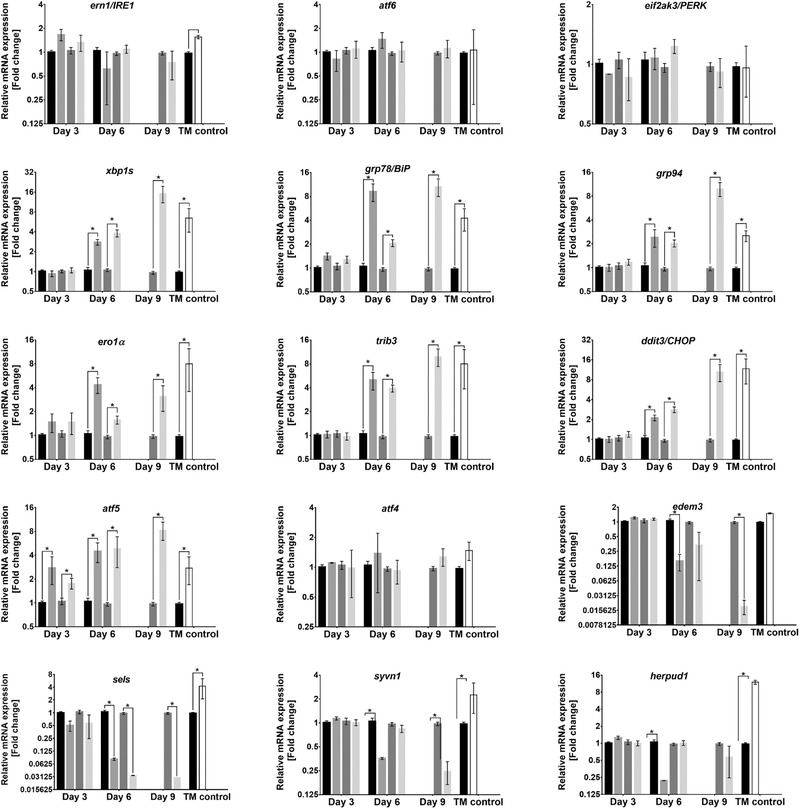
Gene expression profiles of UPR‐ and ERAD‐related genes in the CHO‐hEPO‐Fc cell line during batch and fed‐batch cultures at 37 °C and 32 °C. *ern1/IRE1*, endoplasmic reticulum to nucleus signaling 1 or inositol‐requiring enzyme 1; *atf6*, activating transcription factor 6; *eif2ak3/PERK*, eukaryotic translation initiation factor 2 alpha kinase 3 or protein kinase RNA‐like endoplasmic reticulum kinase; *xbp1s*, x‐box binding protein 1 spliced; *grp78/BiP*, glucose‐regulated protein 78 or binding immunoglobulin protein; *grp94*, glucose regulated protein 94; *ero1α*, endoplasmic reticulum oxidoreductase 1 alpha; *trib3*, tribbles homolog 3; *ddit3/CHOP*, DNA damage inducible transcript 3 or CCAAT‐enhancer‐binding protein homologous protein; *atf5*, activating transcription factor 5; *atf4*, activating transcription factor 4; *edem3*, ER degradation enhancing alpha‐mannosidase like protein 3; *sels*, selenoprotein s; *syvn1*, E3 ubiquitin‐protein ligase synoviolin 1; *herpud1*, homocysteine inducible ER protein with ubiquitin like domain 1. The relative mRNA expression analysis of r‐hEPO‐Fc, calculated using 2‐ΔΔCt method (with 32 °C and 37 °C as treatment and control conditions) and normalized to *gapdh*. The data represents the BC at 37 °C (■), BC at 32 °C (■), FB at 37 °C (■) and FB at 32 °C (■) for day 3, 6 and 9 (only for FB culture). (*) indicated p < 0.05 (t‐test). Experimental values represent the mean of three biological replicates and error bars indicate ± SEM.

Components of the UPR and ERAD pathways are key elements regulating the processing, folding and degradation of proteins within the ER (the main assembly line in the production pipeline) and setting the effectiveness of r‐protein production in CHO cells. Here, we analyzed the gene expression profile of a series of UPR‐ and ERAD‐related targets in response to low temperature stress and assessed how alterations in transcript abundance might contribute to the enhanced r‐protein production under hypothermic growth conditions. These are complex pathways involving a large number of genes, and we chose a gene panel associated with a range of cellular events that occur as a consequence of the accumulation of misfolded proteins in the ER. The target genes had also been shown to respond to ER stress in recombinant CHO cell lines.^[^ ^25^ ^]^ The gene expression profile was analyzed by calculating the ratio of the relative mRNA content of each gene with respect to *gapdh* in low temperature BC and FB cultures using cultures at 37 °C as the un‐treated control condition. After normalization, relative mRNA expression above 1.5‐fold and below 0.58‐fold (with a *t*‐test score of <0.05, Table S2, Supporting Information) was considered as differentially expressed (**Figure** **3**). Treatment of the CHO‐hEPO‐Fc cell line with TM during the exponential phase of the culture was used to chemically induce ER stress and to act as a positive control (TM control) for the comparison with the temperature stress. Since the qPCR‐based ΔΔCt method only provides relative changes in the mRNA expression, the abundance (Ct values) of an additional housekeeping gene (*β‐actin*) was analyzed and compared among all conditions as quality control (Figure S2, Supporting Information). No significant changes in Ct values of *gapdh* and *β‐actin* were observed in all of the conditions studied. Therefore, changes in the relative mRNA expression are mainly associated with changes in the Ct values of the gene targets under evaluation.

The expression of mRNA for ER transmembrane sensors (*ern1/IRE1, atf6, eif2ak/PERK*) and the UPR transactivator *(atf4*) were not altered in response to temperature decrease or TM treatment at mRNA level (Figure 3). This response was as expected for these targets since their regulation mainly occurs at post‐transcriptional level, where phosphorylation plays a key role in their functional regulation.^[^ ^47^ ^]^ Under the same conditions, another subset of genes involved in the UPR and ERAD pathways was significantly affected by the temperature down‐shift (Figure 3 and Table S2, Supporting Information). Upregulation of genes coding for UPR transactivators *xbp1s, ddit3*, and *atf5* as well as ER resident proteins *grp78* (also termed BiP)*, grp94*, *ero1α*, and *trib3* was observed by day 6 and 9 (only for FB), while no significant changes were observed by day 3 for both BC and FB. This gene subset also showed increased mRNA expression when the CHO‐hEPO‐Fc cell line was treated with TM (TM control in Figure 3). Similar results regarding the expression of *xbp1s, grp78, grp94* have been observed in mammalian cell cultures at low temperature,^[^ ^16^, ^21^, ^22^ ^]^ as well as other set of ER‐associated chaperones (not evaluated in this study, e.g., PDIs, Calr, or Canx) that showed similar activation in UPR. In contrast, decreased culture temperature had opposite effects on mRNA amounts for genes related to the ERAD pathways (*edem3, sels, syvn1*, and *herpud1*), which were significantly downregulated by the end of cultures (day 6 in BC and day 9 in FB). The dowregulation of *edem3, sels, syvn1*, and *herpud1* in low temperature cultures showed an opposite trend to their response to TM control, which resulted in a significant increase in the amounts of these mRNAs. In CHO cells, *herpud1* mRNA expression exhibited a time‐dependent response to low temperature.^[^ ^21^ ^]^ However, no further analysis of the mRNA expression in low temperature cultures at late growth phase has been reported. The differential response of sub‐sets of genes to temperature and TM was unexpected. No previous studies have undertaken a similar comparison and there have been no detailed analyses of the regulatory pathways that modulate expression of any of these individual genes at low temperature. Therefore, TDS seemed to cause a mild ER stress, resulting in an increased capacity for protein folding and quality control (by upregulating genes involved in the IRE1/XBP1s branch without activation of the PERK/ATF4 branch of the UPR), and a decreased potential for protein degradation (by downregulating genes related to ERAD pathways). Furthermore, a common pattern was observed in both upregulated and downregulated genes, in which the gene targets showed no changes at day 3 (with the exception of *atf5*), but significant changes at day 6 (for BC and FB cultures) and day 9 (for FB cultures). This indicates that transcriptomic changes and setting of the hypothermic phenotype in the CHO‐hEPO‐Fc cell line took place at least 24 h after the temperature shift, probably a time when a selective mRNA stability/degradation occurs during sustained hypothermic growth.^[^ ^19^ ^]^

To analyze the relationship between the upregulated targets of the UPR and production of the r‐protein, statistical correlations (Pearson) were calculated for the relative mRNA expression of these targets and the hEPO‐Fc production ratio (32 °C versus 37 °C) and cell specific productivity ratio (32 °C versus 37 °C) in both BC and FB cultures . Significantly strong Person's correlations were found between the *q*_hEPO‐Fc_ and the gene expression of *grp78*, *ero1α*, and *trib3* (Figure S3, Supporting Information), indicating that upregulation of mRNA encoding these three proteins correlates with productive CHO cell culture.

## 4 Discussion

The lack of a robust secretory phenotype makes industrially‐relevant CHO cell hosts less than ideal for handling a high recombinant gene trafficking load (at polypeptide and, potentially, RNA level) or for provision of the complex processing requirements (e.g., folding, glycosylation and/or phosphorylation) required for some secreted r‐proteins.^[^ ^4^ ^]^ In the industrial context, a decrease in culture temperature has been successfully applied to increase both specific and volumetric productivities of CHO cells for different therapeutic r‐protein models (e.g., IFNγ,^[^ ^14^ ^]^ anti‐TNFα^[^ ^16^ ^]^). However, the molecular mechanisms that underpin improved specific productivity remain unclear. In this study, we investigated the consequences of lowered culture temperature on the RNA profile for UPR and ERAD components and the relationship of altered expression of specific components to secreted DTE protein model. As expected, a temperature down‐shift decreased the peak of cell density, improved viability (with increased culture lifespan), arrested cell cycle in G1/G0 phase and increased hEPO‐Fc mRNA stability and production (Figures 1 and 2). As part of the hypothermic phenotype, cells exhibited differential expression of genes involved in the secretory and protein degradation pathways, related to the regulation of the ER stress at molecular level (Figure 3).

**Figure 4:**
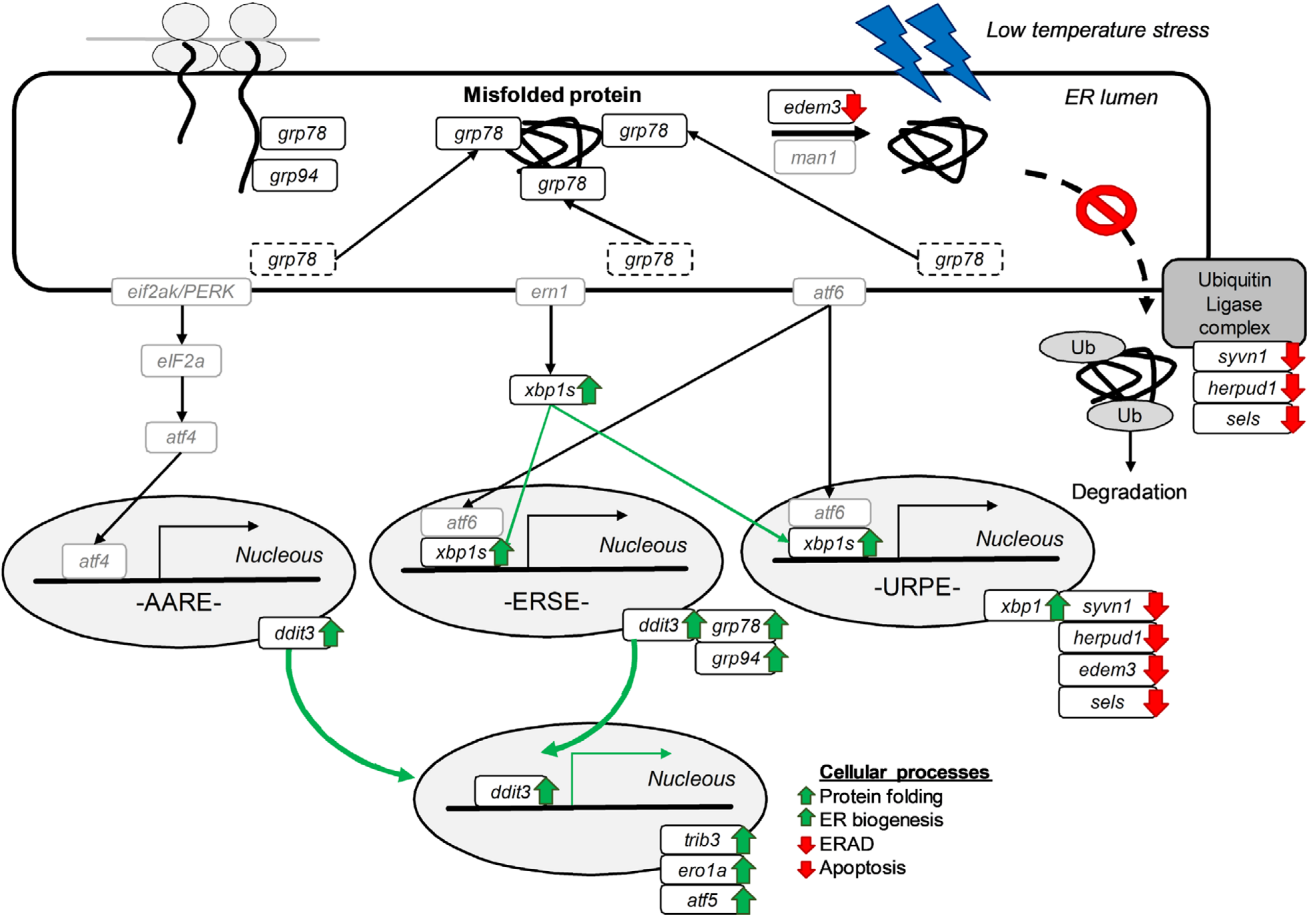
Proposed schematic illustration of the consequences of low temperature stress on the gene expression of the protein folding and processing machinery of the CHO‐hEPO‐Fc cell line. This Figure illustrates the change in the gene expression due to the temperature stress. A green and red arrows (next to each target) represent upregulated and downregulated genes due to the TDS, respectively, while grey boxes indicated no significant changes in the mRNA levels.

This study showed that exposure to cold stress increased the expression of regulatory transcriptional factors and ER‐associated chaperones, particularly those genes involved in the IRE1/XBP1s branch of the UPR. Previous studies with high r‐CHO cell producers have observed similar relative patterns in the expression of UPR markers, which their mRNA abundance (e.g., *grp78*, *grp94*, and *xbp1s*) were highly correlated with r‐protein titers.^[^ ^38^, ^39^ ^]^ Elevated amounts of *grp78* mRNA was associated with the extent of intracellular stress conditions, and therefore, it is no surprise to find high *grp78* expression at late culture stages.^[^ ^40^ ^]^ Antibody‐producing plasma cells are characterized by high expression of *grp78* and *grp94*, linked to immunoglobulin biosynthesis and folding.^[^ ^41^ ^]^ ER chaperones expression is controlled by several UPR‐specific transcription factors, such as ATF6, ATF4, and XBP1s, that generate a series of feed‐back loop regulation.^[^ ^42^ ^]^ During ER stress, activation of XBP1s splicing induces expression of *grp78* mRNA along with other ER specific proteins.^[^ ^43^ ^]^ XBP1s also increases expression of ddit3/CHOP (in a PERK/ATF4 independent manner).^[^ ^44^ ^]^ These reports are consistent with data presented in the current study with elevated expression of ddit3/CHOP and subsequent downstream targets (*atf5, trib3*, and *ero1a*) when *xbp1s* mRNA levels were high at low temperature (Figure 3). In low temperature cultures, increased expression of *xbp1s* was mirrored by increased expression of chaperones shown to be under control of functional XBP1s (*grp78* and *grp94)* and of ddti3/CHOP targets (Figures 3 and **4**). Increased *xbp1s* and *grp78* mRNA expression has been also observed in several r‐CHO cell lines under cold stress, conditions associated with increased cell‐specific productivities.^[^ ^16^, ^21^, ^24^ ^]^ The strong correlations between the abundance of transcripts encoding UPR transactivators/ER chaperones and for the amount of *q*_hEPO‐Fc_ secretion fit well with our expectations of factors that determine the protein secretory capabilities in CHO cell systems (Figure S3, Supporting Information). Although the close relationship between UPR components and productivity in r‐CHO cells has been a rationale for targeting cell engineering strategies,^[^ ^45^ ^]^ such strategies have not yielded guaranteed or reproducible success, highlighting the complexity of the interactions between components in the translation/processing/secretion compartment of the CHO cell.

Increased r‐protein production often pushes the limits of ER folding capacity and elicits the activation of the ERAD pathways to degrade the excess of (abnormally folded/processed) proteins within the ER.^[^ ^46^ ^]^ The contrasting responses of mRNA encoding the set of ERAD markers in low temperature and TM stress was unexpected, particularly in light of the manner in which both stresses provoked similar activation of the UPR sub‐set of mRNA targets. TM treatment upregulated the mRNA expression of *edem3*, *herpud1*, *sels*, and *syvn1* after 6 h of treatment (Figure 3). This response was consistent with previous studies using TM or TG, provoking an upregulation of mRNA encoding ERAD‐specific genes.^[^ ^25^, ^26^ ^]^ Meanwhile, lower culture temperature decreased the mRNA expression of this sub‐set of ERAD markers, suggesting differential regulatory mechanisms that allow discriminatory responses to temperature. TM induces ER stress in cells by inhibition of the first step in the biosynthesis of N‐linked glycans in the proteins, an event that results in many misfolded protein species.^[^ ^47^ ^]^ Activation of *edem3* and ubiquitin ligase complex elements (*herpud1*, *sels*, and *syvn1*) that are key stages for the effective identification of “terminally misfolded” glycoproteins (leading to entry into proteasome‐mediated degradation) can be thought of as a response to a sudden increase in the amount of misfolded proteins after TM treatment. *edem3* and ubiquitin ligase complex elements (*herpud1*, *sels*, and *syvn1*) aid in the recovery of ER homeostasis. Although lowered culture temperature significantly affected N‐linked glycosylation by presenting less processed glycan structures on the mAb constant region^[^ ^34^ ^]^ and activates the UPR at the same extent as TM (Figure 3), it does not drastically lead to elevated amounts of misfolded proteins to trigger similar response as TM and increase ERAD activity. An overall decrease in protein synthesis, observed during low temperature conditions,^[^ ^48^ ^]^ has been verified by two independent transcriptome analyses showing significant decreased expression of genes involved in protein synthesis in mammalian cells.^[^ ^21^, ^22^ ^]^ Data in the present paper shows increased amounts of *ddit3/chop* mRNA in lower temperature conditions (Figure 3), and elevated expression of *ddit3/chop* mRNA has been associated with selectively decreased protein synthesis as a mechanism to restore ER homeostasis during stress.^[^ ^49^ ^]^ Such a decrease in protein synthesis might be associated with decreased protein turnover typically observed in mammalian cells under cold stress.^[^ ^48^ ^]^ A diminished protein trafficking may lead to decreased overload of the folding machinery and lessened amounts of misfolded proteins, explaining why ERAD pathways are downregulated in low temperature cultures. Similar observations have been made in low temperature batch and steady‐state chemostat cultures where CHO cells showed a decreased abundance of mis/unfolded r‐protein and lower flux toward degradation.^[^ ^50^ ^]^ CHO cells also a significantly decreased production of secreted host cell proteins (HCP) in low temperature cultures, indicating that secreted protein pools are also affected by decreased protein translation.^[^ ^32^, ^51^ ^]^ Post‐translational modifications also represent another edge in the regulation of the molecular events occurring in the ER. For instance, acetylation or deacetylation mediated by p300 and SIRT1, respectively, have shown to be key in the modulation of the XBP1s activity.^[^ ^52^ ^]^ Changes in p300 and SIRT1 (or other unknown players) levels under hypothermic conditions might also contribute to the differential response of the UPR and ERAD. Hence, CHO cells at lowered culture temperature seems to respond to several physiological changes to decrease protein trafficking (by decreased protein synthesis) and increased folding capacity (by increased ER chaperone abundance). These results also suggest that the alteration/control of the ERAD pathways (particularly in highly productive culture time points) may offer a potential strategy to enhance r‐protein production in CHO cell factories, although modification of degradation pathways may also have wider implications for the control of overall cellular proteostasis.

Nguyen et al. performed a bioinformatics analysis of genes regulated by temperature and identified several cold‐induced promoters that showed increased activity at lower temperature. The CMV promoter (which is one of the most used for the r‐protein expression and used in the current study) exhibited an increased promoter activity at low temperature and a fourfold and 1.7‐fold increase in transient and stable expression studies with r‐genes.^[^ ^37^ ^]^ These observations are in the line with the r‐gene expression data presented in the current work (Figure 2C,D) and offers a molecular explanation why lowered culture temperature generally induces an increased expression of r‐genes. In conclusion, a lowered temperature contributes to a better secretory phenotype in CHO cells by modifying the expression of genes involved in the UPR and ERAD pathways. Upregulation of UPR‐specific transcriptional activators and ER‐resident proteins suggested an enhanced folding and processing machinery of proteins, whilst downregulation of ERAD components indicated that CHO cells presented a decreased protein degradation under low temperature stress (Figure 4). A deeper understanding of the cellular mechanism to cope with the ER stress in the bioprocessing context presents a unique opportunity for the development of robust CHO cell lines with improved performance in cultures, particularly for the production of DTE proteins.

## Conflict of Interest

The authors declare no conflict of interest.

## Supporting information

Supporting information
