# Supplementary material for: Temperature Down‐Shift Modifies Expression of UPR‐/ERAD‐Related Genes and Enhances Production of a Chimeric Fusion Protein in CHO Cells
Source: Biotechnol J. 2020 Apr 27;16(2):2000081. doi: 10.1002/biot.202000081 (PMC11475507; doi:10.1002/biot.202000081)
Supplement: Supplementary file 1 — Supporting information [file BIOT-16-2000081-s001.pdf]

Supplementary files

Temperature down-shift modifies expression of UPR-/ERAD-related genes and enhances production of a chimeric fusion protein in CHO cells

Mauro Torres[1],

Samia Akhtar[1],

Edward A. McKenzie[1,2],

Alan J. Dickson[1,*]

[1]Manchester Institute of Biotechnology, Faculty of Science and Engineering, University of Manchester, Manchester, UK

[2]Protein Expression Facility, Manchester Institute of Biotechnnology, Faculty of Life Sciences, University of Manchester, Manchester, UK

*Corresponding author: Prof. Alan J. Dickson

Correspondence: Prof.. Alan J. Dickson, Manchester Institute of Biotechnology, Faculty of Science and Engineering, University of Manchester, 131 Princess Street, Manchester M1 7DN, UK. **E-mail**: alan.dickson@manchester.ac.uk

Keywords: Biopharmaceuticals, Chinese hamster ovary (CHO) cells, Transcriptome analysis, Unfolded protein response, Low temperature cultures.

Table S1. Primer details for gene expression analysis.

Symbol	Gene name	Accession no.*	Primer	Sequence (5’-3’)
gapdh	Glyceraldehyde-3-phosphate dehydrogenase	NM_001244854.2	Forward	GCCAAGAGGGTCATCATCTC
			Reverse	CCTTCCACAATGCCAAAGTT
hEPO-Fc			Forward	AGTGCTGAGAGGACAGGCTC
			Reverse	CAGGAAGTTGGAGTACACCCG
Ern1/IRE1	ER to nucleus signaling 1	XM_027436202.1	Forward	AAGGCTGCCCAGTTGTCTAC
			Reverse	ACCGGAAGAGCCTACAGCTA
Atf6	Activating transcription factor 6	XM_003506878.4	Forward	TGCTCATGGGGTTTCCAACA
			Reverse	TCATGCTCTGACCCCACAAC
Eif2ak3/PERK	Eukaryotic translation initiation factor 2 alpha kinase 3	XM_027437608.1	Forward	ACCCTCACTTTTTGCCAGGA
			Reverse	GCAGATGGGTCAAGTGCCTA
Xbp1s	X-box binding protein 1	NM_001244049.1	Forward	CTCGCTTGGGAATGGATGTG
			Reverse	GGTAGACCTCTGGGAGTCC
Grp78/BiP	Heat shock protein family A (Hsp70) member 5	NM_001246739.2	Forward	TCAGCCAATTATCAGCAAACTC
			Reverse	ACAGCTCTAGCAGATCAGTG
Grp94	Heat shock protein 90 beta family member 1	XM_003505850.4	Forward	AGGAAAACCGGGAAGCAACA
			Reverse	GCCCGTTTGGTATGCTTGTG
Ero1α	ER oxidoreductase 1 alpha	XM_007651327.3	Forward	CGCTGCTTCTGTCAGGTTAGT
			Reverse	GCCTTCTGGGTTTCCTCACT
Trib3	Tribbles pseudokinase 3	XM_007652053.3	Forward	TCTCCCCTCCACAGACTAGC
			Reverse	AACCATACAGCCCCAACTCG
Ddit3/CHOP	DNA damage inducible transcript 3	XM_007648093.3	Forward	CACCATACCTGAAAGCAGAA
			Reverse	ACCTCCTGCAGATCCTCATA
Atf5	Activating transcription factor 5	XM_003510884.3	Forward	TCCTAGTCCTGCCATCACCA
			Reverse	GGGCCTTGTACACCTCGATT
Atf4	Activating transcription factor 4	NM_001246812.1	Forward	CAGGTTGCCCCCTTTACAT
			Reverse	CAGGCTTCCTGTCTCCTTCA
Edem3	ER degradation enhancing alpha-mannosidase like protein 3	XM_007641575.3	Forward	CACCTCAAAGATGGGCGAGT
			Reverse	AGATCCAGCCCAAACTGAGC
Sels/VIMP	Selenoprotein S	NM_001256848.1	Forward	CCAGCTACGGCTGGTACATC
			Reverse	GCTAGAGCCTCTTGTCGCTT
Syvn1	Synoviolin 1	XM_016963602.2	Forward	GTCTGCGCAACATACACACA
			Reverse	CGGGAGGCTTTTCAGCTTCA

Herpud1	Homocysteine inducible ER protein with ubiquitin like domain 1	XM_003503612.3	Forward<br>Reverse	TGCATCATGTCGGGTGGTTT<br>ATGATCTGGAGGGAGGCTGT

*Transcript accession number according to CHO-K1 genome assembly released in 2018 (Rupp et al. 2018).

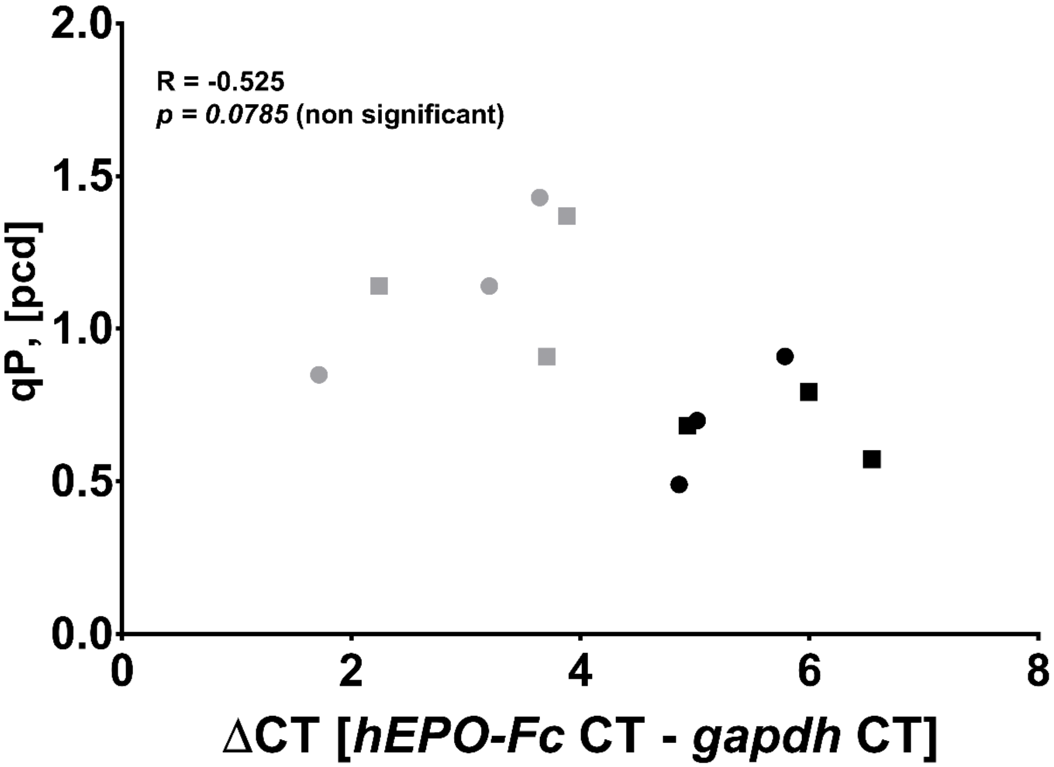

Figure S1. Relationship between ΔCt values for the recombinant hEPO-Fc gene expression and cell specific productivity ($q_{hEPO-Fc}$) in batch (*circles*) and fed-batch (*squares*) cultures at 37°C (*black*) and 32°C (*grey*). The ΔCt values correspond to the difference of Ct values of hEPO-Fc and *gapdh* (housekeeping gene). High abundance of the recombinant gene results in low Ct values for hEPO-Fc and therefore, low ΔCt values indicate higher expression of the hEPO-Fc. Each ΔCt and qP values represent a biological replicate for batch and fed-batch cultures, both at 37°C and 32°C.

Table S2. Statistical analysis of the mRNA expression levels of UPR gene targets

		BC32 vs BC37		FB32 vs FB37	
		t-test	Adjusted p-value	t-test	Adjusted p-value
ern1/IRE1	D3	2.452	0.071	0.996	0.365
	D6	0.974	0.385	0.61	0.948
	D9	n.r.	n.r.	1.067	0.34
atf6	D3	0.361	0.811	0.115	0.913
	D6	0.804	0.715	1.16	0.672
	D9	n.r.	n.r.	0.112	0.936
eif2ak/PERK	D3	1.363	0.214	1.013	0.356
	D6	0.134	0.989	1.071	0.374
	D9	n.r.	n.r.	0.976	0.246
xbp1	D3	0.770	0.484	0.513	0.634
	D6	6.418	**0.003**	6.048	**0.004**
	D9	n.r.	n.r.	3.317	**0.029**
grp78/BIP	D3	2.887	**0.044**	2.190	0.093
	D6	3.564	**0.024**	5.171	**0.006**
	D9	n.r.	n.r.	3.623	**0.022**
grp94	D3	0.034	0.974	1.759	0.153
	D6	2.341	0.079	5.081	**0.007**
	D9	n.r.	n.r.	3.799	**0.019**
ero1α	D3	1.224	0.288	1.061	0.348
	D6	3.442	**0.026**	3.111	**0.035**
	D9	n.r.	n.r.	1.914	0.128
trib3	D3	0.311	0.771	0.237	0.824
	D6	3.201	**0.032**	7.452	**0.001**
	D9	n.r.	n.r.	3.594	**0.022**
ddit3/CHOP	D3	0.038	0.971	1.651	0.174
	D6	5.310	**0.006**	6.466	**0.003**
	D9	n.r.	n.r.	3.124	**0.035**
atf5	D3	1.665	0.171	2.802	**0.048**
	D6	2.788	**0.049**	1.877	0.133
	D9	n.r.	n.r.	3.409	**0.027**
Atf4	D3	10.370	**0.000**	0.016	0.988

	D6	0.459	0.699	0.288	0.787
	D9	n.r.	n.r.	1.089	0.337
edem3	D3	3.704	**0.021**	2.363	0.077
	D6	14.311	**0.000**	2.403	0.074
	D9	n.r.	n.r.	159.21	**0.000**
sels	D3	3.645	**0.022**	1.342	0.251
	D6	183.4	**0.000**	2885	**0.000**
	D9	n.r.	n.r.	8015	**0.000**
herpud1	D3	2.931	**0.042**	0.038	0.971
	D6	666.1	**0.000**	0.134	0.899
	D9	n.r.	n.r.	1.431	0.251
syvn1	D3	2.482	0.068	0.095	0.928
	D6	61.21	**0.000**	1.784	0.149
	D9	n.r.	n.r.	9.553	**0.000**

n.r.: non-reported

highlighted values represent values significantly different.

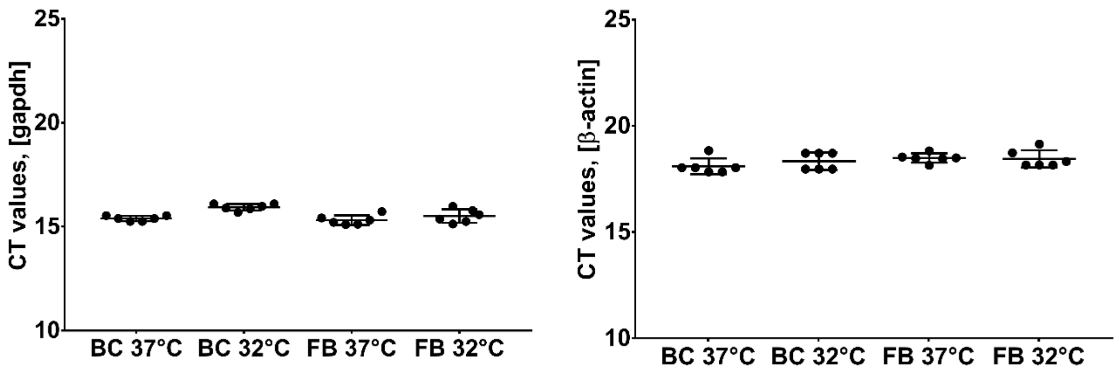

Figure S2. Ct values of the housekeeping genes *gapdh* and *β-actin* of the CHO-hEPO-Fc cell line in batch (BC) and fed-batch (FB) cultures, both at 37ºC and 32ºC. This figure illustrates that the mRNA expression of the internal standard (*gapdh*) gene was similar among all culture conditions.

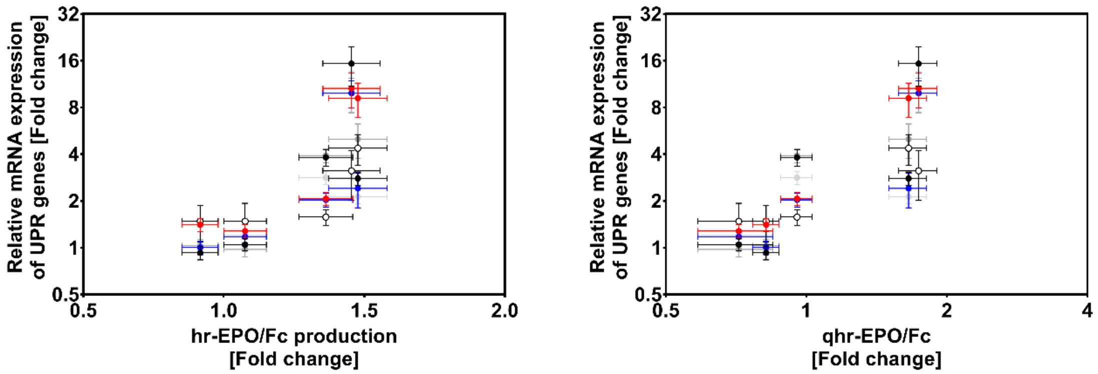

Pearson's correlation		
	mRNA expression vs hEPO-Fc production	mRNA expression vs $q_{hEPO\text{-}Fc}$
xbp1s	R = 0.589 (p = 0.295)	R = 0.712 (p = 0.177)
grp78	R = 0.791 (p = 0.110)	**R = 0.992 (p = 0.0008)**
grp94	R = 0.576 (p = 0.309)	R = 0.744 (p = 0.149)
ero1a	R = 0.748 (p = 0.146)	**R = 0.911 (p = 0.031)**
trib3	R = 0.807 (p = 0.098)	**R = 0.896 (p = 0.045)**
ddit3	R = 0.576 (p = 0.309)	R = 0.702 (p = 0.186)

Figure 3S. Relationship between the mRNA expression of UPR-related targets and hEPO-Fc production (left) and cell specific productivity (right) in low temperature cultures. *Xbp1s* (*black*), *grp78* (*red*), *grp94* (*blue*), *ero1a* (*white*), *trib3* (*dark grey*), *ddit3* (light *grey*). The mRNA expression of upregulated gene targets (i.e., *xbp1s, ddit3*, *atf5*, *grp78, grp94, ero1α* and *trib3*) were plotted against the relative hEPO-Fc production (i.e., titre at 32°C vs titre at 37°C) and the relative cell specific productivity (i.e., $q_{hEPO\text{-}FC}$ at 32°C vs $q_{hEPO\text{-}FC}$ at 37°C) for day 3, 6 and 9 (only for FB cultures). Pearson's correlations were calculated considering each gene target and the relative titer or $q_{hEPO\text{-}FC}$ corresponding to the specific time point and culture mode.

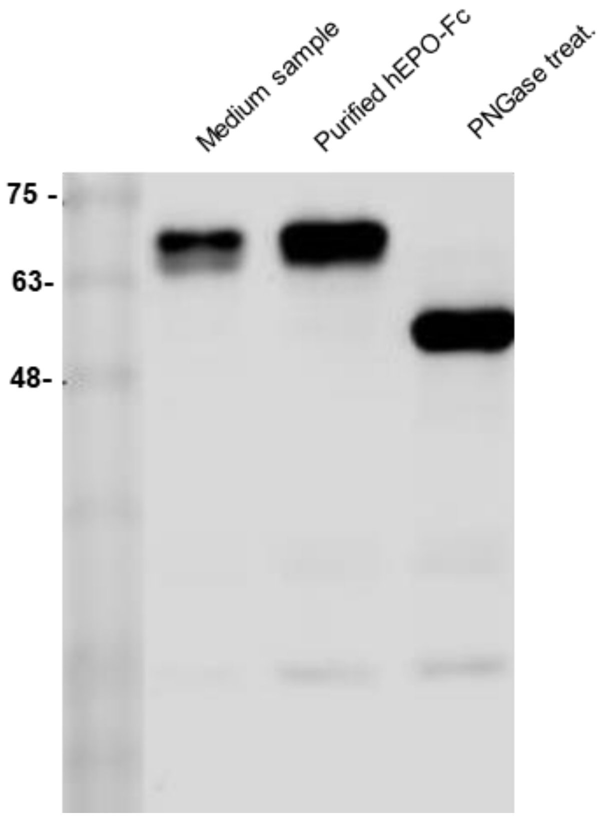

Figure 4S. PNGase treatment to hEPO-Fc. Western blot indicated medium sample from day 6 of batch cultures at 37°C (Lane 1), purified hEPO-Fc using protein A agarose beads (Lane 2) and PNGase treated hEPO-Fc (Lane 3). PNGase treatment was performed by incubating overnight 5 units of enzyme at 37°C.
